# What motivates individuals to volunteer in Ebola epidemic response? A structural approach in Guinea

**DOI:** 10.1186/s12960-019-0409-x

**Published:** 2019-11-01

**Authors:** Lonzozou Kpanake, Togba Dounamou, Paul Clay Sorum, Etienne Mullet

**Affiliations:** 1 University of Québec – TELUQ, 5800, rue Saint-Denis, Bureau 1105, Montréal, Québec H2S 3L5 Canada; 2 Direction Communale de la Santé de Matoto, Wanindara 1, Commune de Ratoma, Conakry, Guinea; 3 Albany Medical College, Latham Med-Ped, 724 Watervliet-Shaker Road, Latham, Albany, NY 12110 United States of America; 4 Institute of Advanced Studies (EPHE), 17 bis, rue Quefes, Plaisance du Touch, 31830 Paris, France

**Keywords:** Volunteering, Motives, Ebola, Epidemic response, Africa

## Abstract

**Background:**

The 2014–2016 Ebola epidemic in West Africa placed greater demands on the affected countries’ already scarce health workforce. Consequently, governments in the most affected West African countries made appeals for volunteers to join Ebola response programs. Those volunteers played an important yet high-risk role in aiding the victims of the Ebola epidemic and in limiting its spread. However, little is known as to what motivated those volunteers to commit themselves to the Ebola response programs. This information is important for planning for volunteer recruitment strategies during future epidemics. The aim of the present study, therefore, was to identify and assess the motivations that led individuals to volunteer for Ebola response programs in West Africa.

**Methods:**

The study participants were 600 persons who volunteered through the Guinean Ebola response program during the 2014–2016 epidemic. From February to May 2016, they were presented with a questionnaire that contained 50 assertions referring to possible motives for volunteering in the Ebola response program and indicated their degree of agreement with each of them on a scale of 0–10. The responses were analyzed using factor analysis.

**Results:**

Seven separable volunteer motivations were identified. “Feeling of patriotic duty” (*M* = 9.02) and “Feeling of moral responsibility” (*M* = 8.12) clearly emerged as the most important. Second-tier motivations were “Compliance with authority” (*M* = 6.66), “Desire to use one’s skills for a collective good” (*M* = 6.49), “Seeking personal growth” (*M* = 5.93), “Desire to gain community recognition” (*M* = 5.13), and “Hoping for a career reorientation” (*M* = 4.52).

**Conclusions:**

These findings strongly suggest that volunteer recruitment, if needed in future Ebola epidemics, must adopt a multifaceted motivational approach rather than focus on one single motivator. Putting relatively more emphasis on motivational messages referring to patriotic values, as well as to moral responsibility, would likely increase volunteering.

## Background

The 2014–2016 Ebola epidemic in West Africa reached a scale never seen before. Over 28 600 people were infected with Ebola virus, and over 11 000 died [1]. During the epidemic, the characteristics of the virus—the high fatality rate and the high level of transmissibility—combined with the chronically fragile and under-resourced health systems to place greater demands on the affected countries’ already scarce health workforce [2, 3]. As a result, governments in the most affected West African countries—Liberia, Sierra Leone, and Guinea—and a variety of international non-governmental organizations (e.g., Doctors Without Borders, International Red Cross and its national societies, and International Medical Corps) appealed for volunteers to join the Ebola response programs [4–6]. Those volunteers were lay people with diverse occupational backgrounds, including students, teachers, taxi drivers, street vendors, farmers, and artisans [5–7]. They were recruited from and worked in their own communities; as such, they were uniquely positioned to promote protective health behaviors and appropriate healthcare seeking behaviors. They received training for a specific role in the Ebola response programs and were incorporated into the teams of Ebola frontline responders [5–7]. The most common roles undertaken by those volunteers were conducting safe burials of Ebola victims, taking Ebola patients to treatment centers, cleaning Ebola treatment centers, educating the public about Ebola and what to do to prevent it, and assisting in surveillance to detect possible Ebola cases in their communities by, for example, screening people for fever in public places and monitoring Ebola contacts [6–8]. The volunteers’ contributions were critical in the response to the epidemic. For example, Tiffany et al. estimated that volunteers responsible for safely burying Ebola-infected individuals averted between 1411 and 10 452 secondary Ebola infections, reducing the epidemic by 4.9 to 36.5% [9]. The volunteers took great risks for themselves and their families by joining Ebola response programs: frontline responders were 21–32 times more likely to be infected with Ebola than the general population; 815 were infected, among whom 47% died [10]. Although called “volunteers,” they were far removed from a classic definition of a “volunteer”: “a person who does something, especially helping other people, willingly and without being forced or paid to do it” [11]. Indeed, voluntary work in Ebola response program has evolved into a remunerated activity, accompanied by job descriptions and titles. Although these volunteers were not paid a salary, they received some form of remuneration, the amount depending on the activities and the organization, and some volunteers had access to it and some not [12, 13].

Given the absolutely essential role of the volunteers in aiding the victims of the epidemic and in limiting its spread, the high risk associated with volunteering and the certainty that volunteers would be part of the response to future epidemics response, it is critical to understand how best to recruit and retain volunteers. A starting point is to examine the underlying motives that led individuals to volunteer during the recent Ebola epidemic. The present study is the first to address this issue.

Previous studies on what motivates individuals in Africa to volunteer in health programs have mainly used qualitative methods and have consistently found a wide variety of motives. Akintola [14] interviewed 57 volunteer caregivers of people living with HIV/AIDS in South Africa and identified 11 motives: (a) altruistic concerns for others and community, (b) employment and career benefits, (c) the desire by unemployed persons to avoid idleness, (d) an opportunity to learn caregiving skills, (e) an opportunity to put their own skills to good use, (f) personal growth, (g) a means of attracting good things to themselves, (h) a religious calling, (i) the hope of gaining community recognition, (j) a way to deal with a devastating personal experience of AIDS, and (k) an effort to improve society. Takasugi [15] used focus group discussions with 23 volunteer community health workers in Kenya to identify their different motivational drivers: (a) hope that the voluntary role would lead to salaried employment, (b) a desire to help improve the standard of living and health awareness in the community, (c) a means of gaining respect from the community, (d) hope of receiving monetary incentives, (e) hope of gaining valued items, (f) a means of obtaining preferential service when attending a health facility, and (g) an opportunity to learn. Kironde and Klaasen [16] found that volunteers in tuberculosis control programs in South Africa were motivated by (a) hope for eventual remuneration, (b) the desire to work for the good of others, (c) the desire to gain work experience, (d) the need to find something to do with one’s spare time, and (e) the fact that the community-based tuberculosis program was new.

While those previous studies highlight the diversity of motives underlying volunteering in health programs, they cannot help in assessing the strength of each of those motives, in distinguishing among them as a function of their impact on volunteering and in understanding the structural relations among them on the basis of their similarities and differences. Only a few studies have used the structural approach needed to address these issues. The core propositions of this approach are that different volunteers pursue different goals, that the same volunteer may have different motives, that motives to volunteer relate to one another in a coherent and meaningful way that reflects a motivational structure [17], and that the strength of each kind of motive can be assessed in a precise way.

Claxton-Oldfield et al. [18] examined motives for becoming volunteers in hospice palliative care in Canada. They used a 65-item questionnaire that consisted of assertions referring to possible motives for volunteering and identified—based on factor analysis of the data—five different kinds of motives: “altruism” (e.g., volunteering is a way to help others), “civic responsibility” (e.g., volunteering is a way to give back to the community), “personal gain” (e.g., volunteering could help with future goals), “self-promotion” (e.g., volunteering helps to gain attention), and “leisure” (e.g., volunteering is a hobby). “Altruism” (*M* = 21.44 on a scale of 0–25) and “civic responsibility” (*M* = 18.64) clearly emerged as the strongest motivators, while “leisure” (*M* = 14.74), “self-promotion” (*M* = 11.46), and “personal gain” (*M* = 9.46) were second-tier motivators.

Using similar research methods and statistical analysis, Clary et al. [19] investigated the motives of volunteers in the United States of America in services to children, to the families of cancer patients, to the physically handicapped, and to social service and public health clients, as well as in blood services and disaster relief. They found a motivational structure reflecting six different kinds of motives: “to express values related to altruistic concerns for others” (e.g., it is important to help others), “to acquire learning experiences and/or exercise professional skills” (e.g., gaining a new perspective on things), “to strengthen social networks” (e.g., it is important to the people I know best), “to gain career related experience” (e.g., get a foot in the door at a place where one would like to work), “to reduce negative feelings about oneself or address personal problems” (e.g., working through one’s own personal problems), and “seeking personal growth” (e.g., volunteering increases self-esteem). The strongest motives were “to express values related to altruistic concerns for others” (*M* = 5.37 over 7), “to acquire learning experiences and/or exercise professional skills” (*M* = 5.13), and “seeking personal growth” (*M* = 4.64). The impacts of motives involving career opportunity (*M* = 4.54), addressing personal issues (*M* = 3.25), and strengthening personal social networks (*M* = 2.95) were relatively weak.

Finally, Mousa and Freeland-Graves [20] examined the structure of the motives of people in the United States who volunteer in food rescue. They also found a complex motivational structure with four separate motives: “altruistic” (e.g., want to help community), “social life” (e.g., want to decrease one’s loneliness), “career improvement” (e.g., increase career options), and “service requirement” (e.g., required for membership in their social group). The most prevalent motivator was altruism (29%), followed by career improvement (28%) and social life improvement (28%). Those who volunteered to fulfill a requirement had the lowest score (15%).

These three studies that used a structural approach to examine motivations underlying volunteerism focused on contexts in which volunteering activities involved no life-threatening risk and were conducted exclusively in high-income countries in which volunteering conditions are relatively good. No study has yet examined the motivations of volunteers in Ebola epidemic responses, where the high Ebola fatality rate—which varied from 25 to 90% [21], the high level of its transmissibility [21], and the challenging working conditions of Ebola responders in Africa [22]—make the issue of volunteering much more challenging. The present study is the first to use a structural approach to examine volunteerism in an African context. The aim was to delineate the motivational structure underlying volunteering in Ebola response programs and to measure the strength of each kind of motive.

Our first hypothesis, based on previous studies [14–20, 23, 24], was that at least six different kinds of motives for volunteering in Ebola response programs would be found: (1) a feeling of patriotic duty, (2) a feeling of moral responsibility, (3) a desire to use one’s skills for a collective good, (4) compliance with authority, (5) a desire to gain community recognition, and (6) a seeking for personal growth. Our second hypothesis, based on the devastating consequences of Ebola in the affected poor countries in West Africa [1–3], was that the most important motives would be patriotism and feelings of moral responsibility toward fellow citizens and that the contribution of the other motives would be relatively moderate.

## Methods

### Study setting

The study was conducted in Guinea, a West African country with a population of 12 609 000 [25]. Its health system is characterized by a relative shortage of healthcare personnel and a lack of investment in the healthcare infrastructure [26]. It was one of the countries most affected by the 2014–2016 Ebola epidemic, with 3811 cases and 2543 deaths [1]. The World Health Organization reported 199 Ebola infections among the country’s Ebola frontline responders, among whom 55% died [10]. The study site was Conakry, the capital and largest city in Guinea and one of the Ebola epicenters [27].

### Participants

Participants were Guinean citizens who volunteered through the Guinean Ebola response program during the 2014–2016 epidemic and worked in the district of Matoto, Conakry. One co-author, who headed the Ebola response program in Matoto, led the recruitment of participants and the collection of data. In February 2016, his team of five research assistants sent invitations to participate in the study to all the volunteers of the district with full explanations of the study and its procedures. A total of 770 were invited and 77.9% agreed to participate. The 600 participants (421 men and 179 women) provided informed consent. Their age ranged from 18 to 67 years (*M* = 31, *SD* = 9.22). Overrepresentation of men in the sample reflects gender inequalities in Guinean workforce. See Table 1 for more details about their demographic characteristics.

**Table 1:** Demographic characteristics of participants

Characteristics	Levels	Number	Percentage
Gender	Women	179	30
	Men	421	70
Age	18–24	224	37
	25–40	279	47
	+ 40	97	16
Education	Elementary school	32	5
	Middle school	172	29
	High school	233	39
	College	163	27
Religion	Christians	211	35
	Muslims	279	47
	Animists	76	13
	Atheists	43	7
Total		600	100

### Material

The material was a 50-item questionnaire that consisted of assertions referring to reasons that could motivate one to volunteer in Ebola response programs. The items were devised in multiple ways. First, a list of items was created by the investigators on the basis of previous studies on motivations for volunteering in community health programs [14–20, 23, 24]. This list was then shown consecutively to two focus groups of eight volunteers each who, under the direction of one of the authors, reformulated items judged as ambiguous and suggested additional items. The last augmented list was then presented to a third focus group composed of eight other volunteers who found the assertions easy to understand and made no additional suggestions.

Thirty-four of the items are shown in Table 2. The common wording of all items—“One of the reasons I volunteered in Ebola response programs was that …”—was chosen to reflect the fact that several motives can be operating at the same time or at different times for the same person [17]. An 11-point scale was printed following each sentence. The two extremes of the scales were labeled “Disagree completely” (0) and “Agree completely” (10). Using a 0–10 rating scale yields sufficient diversity in the data [28]. The language used throughout the study was French, the official language in Guinea.

**Table 2:** Results of the exploratory factor analysis

One of the reasons why I joined the team was that …	Factor							*M*	*SD*
	I	II	III	IV	V	VI	VII		
… Guinea needed the mobilization of all its citizens.	*.79*	.03	.04	.03	− .02	− .04	.01	9.19	2.47
… fighting against Ebola was a major objective	*.72*	.14	− .06	.05	− .06	− .01	.16	9.06	2.69
… it was a testimony of love of the homeland.	*.71*	.07	.02	− .20	.24	.02	− .06	9.03	2.72
… citizens have the duty to defend their country against all threats.	*.50*	.14	.11	.08	− .03	.01	− .18	8.82	2.86
… I considered it was a moral duty.	.04	*.74*	.16	.09	− .00	.16	.02	7.55	3.84
… I considered it was a duty to the people.	.11	*.66*	.09	.10	.03	.11	− .00	8.17	3.52
… it was a morally rewarding experience.	.13	*.62*	.24	.11	.07	− .11	.02	8.39	3.27
… it was a way to prove to myself that I was a responsible citizen.	.25	*.49*	.20	− .07	.18	.10	− .06	8.37	3.22
… my duty is to respond to Guinean health authorities’ requests.	.00	− .01	*.72*	.09	.02	.13	− .06	6.98	4.15
… it was the way to keep having a good image of myself.	.12	.03	*.69*	.19	− .06	.25	− .02	6.46	4.25
… other people did it and I have deep esteem for them.	.09	.11	*.62*	.16	.03	.11	.17	6.65	4.29
… other many people I know did it.	.03	.13	*.62*	.17	.09	− .01	.10	6.85	4.05
… I wanted to respond to the religious authorities’ call.	− .06	.23	*.60*	− .02	.12	.32	.07	6.97	3.93
… the idea that Ebola could spread to the rest of the country was unbearable.	.06	.25	*.52*	.22	.12	.02	.17	6.58	4.23
… I wished to overcome emotional challenges.	− .02	.31	*.52*	.14	.27	− .12	.00	6.62	4.21
… I thought it could be a transformative experience.	− .13	.22	*.43*	.02	.27	.07	.25	6.16	4.28
… it was a way to broaden my professional experience.	.03	.17	.22	*.71*	.20	.15	.10	6.53	4.23
… I could professionally contribute to the development of the country.	.02	.20	.21	*.70*	.11	.04	.06	6.96	4.08
… I wished to bear testimony of my active commitment to the Guineans.	.02	.02	.15	*.63*	.22	.11	.16	6.55	4.37
… it was an opportunity to make use of my professional expertise.	− .07	− .08	.19	*.57*	.21	.14	− .04	5.93	4.44
… I thought that, by doing so, I could personally benefit.	.00	− .17	.20	− .05	*.68*	.13	− .01	6.08	4.42
… it was an opportunity to improve self-achievement.	− .03	.11	.05	.18	*.63*	.09	.18	5.11	4.46
… it was an opportunity to experience extreme situations	.10	.02	.03	.26	*.57*	.19	.09	5.99	4.39
… it was a way to behave in harmony with myself.	− .00	.19	.11	.31	*.57*	.07	− .07	6.31	4.29
.. it was a kind of personal challenge.	.06	.21	.02	.29	*.55*	.17	.07	6.18	4.42
… it was a mean to attract the sympathy of people in my community.	.01	.01	.10	.04	.13	*.76*	− .02	5.77	4.43
… it was a mean to attract the sympathy of my colleagues.	− .04	.24	.06	.24	− .00	*.66*	.07	5.51	4.54
… my favorite football players have urged the necessity of volunteerism.	− .02	− .10	.13	− .04	.12	*.60*	.13	3.59	4.50
… I wished people like me better.	.02	.11	.05	.21	.23	*.60*	.09	5.49	4.52
… I wished to become a better professional.	− .00	.17	.22	.07	.13	*.43*	.20	5.29	4.63
… I was not convinced that the teams in place were fully competent.	− .03	.08	.00	.12	.04	.08	*.76*	4.58	4.48
…, in some way, I felt forced to do so.	− .10	.09	.13	− .06	.09	− .04	*.65*	5.00	4.63
… my current job was boring.	.05	− .22	.05	.16	− .05	.30	*.60*	3.87	4.42
… the job I had did not offer any true challenge..	.09	− .10	.25	.11	.15	.31	*.56*	4.62	4.40
Explained variance	2.08	2.33	3.38	2.40	2.39	2.53	2.04		
Percent of variance explained	.06	.07	.10	.07	.07	.07	.06		
*M*	9.02	8.12	6.66	6.49	5.93	5.13	4.52		
*SD*	1.88	2.46	2.73	3.18	2.96	3.05	3.14		
*N* ≥ 8 (%)	77	63	39	40	36	26	16		

I = Feeling of patriotic duty, II = Feeling of moral responsibility, III = Compliance with authority, IV = Desire to use one’s skills for a collective good, V = Seeking personal growth, VI = Desire to gain community recognition, and VII = Hoping for a career reorientation

### Procedure

The questionnaire was paper-based. Participants answered individually in a vacant classroom in a local school or the participant’s private home, depending on what was the most convenient. The assistant was not present when the participants filled out the questionnaire (in order not to influence their responses). The questionnaire took approximately 30 min to complete. Full anonymity was provided to all participants. Data collection was carried out from February to May 2016.

Ethics approval for the study was obtained from the Guinean National Review Board for Health Research, the Guinean National Review Board for Research on Ebola, and the Institutional Review Board of the University of Quebec-TELUQ.

### Data analysis

Means and standard deviations were computed for each item and for the whole sample. An exploratory factor analysis was conducted on the raw data to see whether identifiable groups of items emerged that were statistically correlated (factors). The means and standard deviations of the agreement scores of the combined items of each factor were then computed, and the effects of demographic characteristics on scores for each factor were assessed through analyses of variance (ANOVA).

## Results

Item mean scores ranged from 2.87 to 9.19 (out of 10). A first exploratory analysis that was conducted on the whole set of items showed that 16 of them did not load (< .30) on any factor or loaded on more than one factor. These items were removed, and a second exploratory factor analysis was conducted on the 34 remaining items. Using the screen test—a method of deciding how many factors should be retained in a factor analysis—we observed seven interpretable factors with eigen-values ranging from 1.22 to 6.87. They accounted for 50% of the variance. This seven-factor solution was retained and subjected to Varimax rotation—a statistical method that enables to simplify the expression of complex items in order to look for independent factors. Main results are shown in Table 2.

The first factor explained 6% of the variance. It was labeled “Feeling of patriotic duty” since it loaded positively on items expressing the idea that volunteering in response to the Ebola epidemic was considered a civic duty to end the devastating consequences of Ebola in the country. Its mean score was very close to the maximum rating: 9.02 out of 10. Participants with a college degree scored significantly higher (*M* = 9.16) than other participants (*M* = 8.74), *p* < .02. Participants who declared themselves Christians scored significantly higher (*M* = 9.57) than those who declared themselves Muslims (*M* = 8.97), *p* < .02.

The second factor (7% of the variance) was called “Feeling of moral responsibility” because all items expressed the idea that volunteers may have felt a moral responsibility to help their fellow citizens infected with Ebola. The mean score was very high (*M* = 8.12) and significantly higher among participants with a college degree (*M* = 8.29) than among other participants (*M* = 7.79), *p* < .02.

The third factor (10% of the variance) was called “Compliance with authority” because it expressed the idea that volunteers may have joined the Ebola response program in order to fulfill a cultural prescription, to comply with a religious obligation or to fulfill a political requirement. The mean score was slightly above the middle of the agreement scale (*M* = 6.66).

The fourth factor (7% of the variance) was called “Desire to use one’s skills for a collective good” because it expressed the idea that volunteering in the Ebola response program may have been seen as an opportunity to put one’s own professional skills to make a positive impact on the Guinean society. The mean score was also slightly above the middle of the agreement scale (*M* = 6.49), and significantly lower (*M* = 5.48) among participants who declared themselves Christians than among participants who declared themselves Muslims (*M* = 6.69), *p* < .005.

The fifth factor (7% of the variance) was called “Seeking personal growth” because it expressed the idea that volunteering may have been seen as an opportunity to gain personal growth. The mean score was also slightly above the middle of the agreement scale (*M* = 5.93).

The sixth factor (7% of the variance) was called “Desire to gain community recognition” because it expressed the idea that volunteers may have hoped to gain social benefits through volunteering. The mean score was in the middle of the agreement scale (*M* = 5.13) and significantly lower among participants who declared themselves Christians (*M* = 4.39) than among participants who declared themselves Muslims (*M* = 5.21), *p* < .05.

Finally, the seventh factor (6% of the variance) was called “Hoping for a career reorientation” because it expressed the idea that volunteering may be seen as providing more interesting working experience than the current one. The mean score was slightly below the middle of the agreement scale (*M* = 4.52).

## Discussion

The first objective of this study was to delineate the motivational structure underlying volunteering in Ebola epidemic response. Through factor analysis, we have been able to identify and interpret seven separable motivational factors. This finding is consistent with previous research [18–20] suggesting that while volunteering is certainly driven by a great number of particular motives, these motives relate to one another in a coherent and meaningful way that enables the emergence of a factorial structure. The components of this structure are not redundant: When considering motivational factors such as “Feeling of patriotic duty,” “Feeling of moral responsibility,” and “Desire to use one’s skills for a collective good,” we are not looking at the same motives under different guises, but are really considering three different, empirically separable kinds of motives.

The other objective was to measure the importance of each kind of motive to the volunteers. The emergence of a factorial structure enabled measurement of the strength of each kind of motive. As expected, the respondents reported that their most important motive to volunteer was the “Feeling of patriotic duty” (*M* = 9.02 over 10). This finding supports the World Health Organization’s observation during the recent Ebola epidemic in Liberia that “Local volunteers, who worked in treatment centers, on burial teams, or as ambulance drivers, were driven by a sense of community responsibility and patriotic duty to end Ebola and bring hope back to the country’s people” [29]. With the devastating consequences of Ebola in the affected poor countries in West Africa, governments’ Ebola response emphasized patriotic consciousness. Presidents of affected countries presented the epidemic as a threat to the nation’s economic and social fabric [29] and announced several wartime measures including state of emergency declarations, curfews, national border closings, and military deployment [30]. Mass campaigns presented the epidemic as threatening the survival of the country, as illustrated in posters stating “Ebola c’est la guerre” [Ebola it’s a war] and calling for mobilization of all citizens for the “war” against Ebola [31]. Citizens were encouraged to take an active role in the response to the epidemic by adopting and promoting protective behaviors against the virus, while local media portrayed Ebola response as a test of citizens’ patriotism [32].

As also expected, the respondents reported that “Feeling of moral responsibility” was another strong motivator for volunteering, indeed their second-ranked motivation (*M* = 8.12). This motive was already identified as a common reason for volunteering in community health programs in Kenya [15] and Botswana [33]. Committing oneself to conduct safe burials for fellow citizens who died by Ebola, to help those who are infected with Ebola to get access to treatment centers, or to educate the others about what to do to prevent the virus appears to be the way some Guinean people chose to respond to feelings of moral responsibility toward their fellow citizens. While using patriotic rhetoric to encourage citizens’ mobilization for the epidemic response, governments in Ebola-affected countries in West Africa did not resort to mandatory prescriptions. Thus, citizens who volunteered were free moral agents who were truly willing to help. Those volunteers may have felt they were personally concerned with the epidemic since the victims were their family members, their friends, their neighbors, or at least their fellow citizens. This concern for the victims may have strengthened the volunteers’ sense of group belonging in the face of Ebola and increased their feelings of moral duty to join the epidemic response program. This interpretation is in accord with the Common Ingroup Identity Model [34] which suggests that prosocial intergroup responses increase when members of different groups (e.g., patients infected with Ebola vs volunteers) can see themselves as part of the same group.

The third most highly rated factor was “Compliance with authority” (*M* = 6.66). This finding is corroborated by those of previous studies [14, 33, 35]. Volunteers in community health were motivated by calls from religious leaders in South Africa [14], traditional chiefs in Uganda [35], and the government in Botswana [33]. Influential community figures such as traditional chiefs, spiritual authorities, and political leaders played major roles in efforts to motivate people to volunteer for the Ebola response programs in Guinea and the other affected African countries [36].

“Desire to use one’s skills for a collective good” was the fourth most highly rated factor (*M* = 6.49). This motive has been reported by volunteers involved in HIV/AIDS care in South Africa [14] and in an immunization program in Uganda [37]. In a context of high unemployment rates in Ebola-affected African countries [38], involvement in volunteering activities may be seen as an opportunity for unemployed persons to make use of their professional skills.

The fifth most highly rated factor was “Seeking personal growth” (*M* = 5.93). This motive was identified in Takasugi et al.’s work on motivations for volunteering in community health in Kenya [15]. Previous studies suggest that volunteering has impacts on the lives of the volunteers, including personal growth, an increase in self-esteem, and learning how to keep things in perspective [39, 40].

“Desire to gain community recognition” was the sixth most highly rated factor (*M* = 5.13). Consistent with this finding, community health volunteers in South Africa [14], Kenya [15], and Uganda [35] have reported that their commitment to volunteering was driven by a hope of gaining community recognition. Some volunteers in Uganda acknowledged that volunteering helps them to take up roles in local government [35], while Arab women volunteering in the Women’s Health Program in Israel indicated significant positive changes in their social status [41].

Finally, “Hoping for a career reorientation” (*M* = 4.52) was also an identifiable motive but had the least impact on volunteering. This motive was previously reported by community health volunteers in South Africa [14]. Owing to the high unemployment rate and poverty in Guinea, many Guineans may have felt coerced to take jobs that were below their professional skills. Those workers were likely not making full use of their potential and may have seen volunteering as an opportunity to help them to reorient their careers.

While the study provides important perspectives about motives for volunteering for Ebola epidemic response, a number of limitations highlight the need for caution in interpreting the data. First, the sample was composed of Ebola response volunteers who lived and worked in one specific area during the epidemic. This study’s results must, therefore, be generalized with care to volunteers from other areas of the country or other parts of Africa. Second, while the use of a self-reported measure enabled us to find a clear motivational structure that can be easily replicated, this might have introduced social desirability bias in the responses if participants wanted to claim socially desirable values and deny socially undesirable ones. However, this seems unlikely because the participants knew that their answers were completely anonymous and studies have shown that allowing people to answer questions completely anonymously decreases a person’s motivation to distort reports in socially desirable directions [42, 43]. That said, the present findings should be regarded as preliminary and future research should verify these results using methods that excel completely in preventing social desirability bias. Third, the researcher did not ask further questions to elucidate the reasons underlying respondents’ ratings. This study would be strengthened by follow-up studies using qualitative methods that sought to further elucidate the reasons underlying each of the identified motives. Fourth, the lack of information about participants’ professional background did not enable to assess the veracity of motives referring to “Desire to use one’s skills for a collective good.” Finally, given that volunteerism in Ebola response program has evolved into a remunerated activity, it seems likely that gaining financial benefits has been a motivator among many volunteers.

## Conclusions

This study sought to identify and assess the motivations behind volunteering for Ebola response programs in Guinea. Its findings are the first that could help inform policymakers in Guinea and probably in other African countries, about how to devise and organize strategies for the recruitment and retention of volunteers during future Ebola epidemics. The structural approach helps shed light on the various motives of the volunteers and appreciate the strength of each of those motives. The results showed that each of the identified motives had a significant impact on volunteering. These findings strongly suggest that volunteer recruitment strategies must be multifaceted rather than focused on one single motivator. When implementing those strategies, policymakers should, however, put relatively more emphasis on motivational messages referring to patriotic values, as well as to moral responsibility toward fellow citizens. At an individual level, recruiters may be able to use the motives identified in this study to detect which ones would drive a particular individual and to adapt their recruitment messages accordingly. Finally, while strategies presented above may help increase volunteerism, policymakers must also take into account the reality that adequate expectation management regarding remuneration or incentives is essential, to sustain motivation [44].

## Data Availability

All data collected is available and can be accessed by contacting the corresponding author.

## Abbreviations

AIDS: Acquired immunodeficiency syndrome
ANOVA: Analysis of variance
HIV: Human immunodeficiency virus
M: Mean
SD: Standard deviation

## References

[1] World Health Organization (2016). Ebola situation report – 30 March 2016.

[2] Shoman H, Karafillakis E, Rawaf S (2017). The link between the West African Ebola outbreak and health systems in Guinea, Liberia and Sierra Leone: a systematic review. Global Health.

[3] Barbiero VK (2014). It’s not Ebola … its’s the systems. Glob Health Sci Pract.

[4] BBC News (2014). Volunteer Ebola health worker: “I’m not crazy, just brave enough”.

[5] Perry HB, Dhillon RS, Liu A, Chitnis K, Panjabi R, Palazuelos D (2016). Community health worker programmes after the 2013-2016 Ebola outbreak. Bull World Health Organ.

[6] Miller NP, Milsom P, Johnson G, Bedford J, Kapeu AS, Diallo AO (2018). Community health workers during the Ebola outbreak in Guinea, Liberia, and Sierra Leone. J Glob Health.

[7] The Guardian (2016). Volunteers who fought Ebola for Sierra Leone – one year on.

[8] GOV. UK, Department for International Development (2015). Ebola heroes: meet the ordinary people doing extraordinary work to defeat the disease in Sierra Leone.

[9] Tiffany A, Dalziel BD, Kagume Njenge H, Johnson G, Nugba Ballah R, James D (2017). Estimating the number of secondary Ebola cases resulting from an unsafe burial and risk factors for transmission during the West Africa Ebola epidemic. PLoS Negl Trop Dis.

[10] World Health Organization (2015). Health worker Ebola infections in Guinea, Liberia and Sierra Leone: a preliminary report 21 May 2015.

[11] Cambridge Dictionary. Volunteer. Retrieved from: https://dictionary.cambridge.org/fr/dictionnaire/anglais/volunteer. Accessed 12 July 2019.

[12] Nunes J (2016). Ebola and the production of neglect in global health. Third World Q.

[13] Nunes J. Doctors against borders: Médecins Sans Frontières and global health security. In: Hofman M, Au S, editors. The politics of fear: Médecins Sans Frontières and the West African Ebola epidemic. Oxford: Oxford University Press; 2017. p. 3–24.

[14] Akintola O (2011). What motivates people to volunteer? The case of volunteer AIDS caregivers in faith-based organizations in KwaZulu-Natal, South Africa. Health Policy Plan.

[15] Takasugi T, Lee ACK (2012). Why do community health workers volunteer? A qualitative study in Kenya. Public Health.

[16] Kironde S, Klaasen S (2002). What motivates lay volunteers in high burden but resource-limited tuberculosis control programmes? Perceptions from the northern Cape province, South Africa. Int J Tuberc Lung Dis.

[17] Apter MJ (2001). Motivational styles in everyday life: a guide to Reversal Theory.

[18] Claxton-Oldfield S, Wasylkiw L, Mark M, Claxton-Oldfield J (2011). The inventory of motivations for hospice palliative care volunteerism: a tool for recruitment and retention. Am J Hosp Palliat Care.

[19] Clary EG, Snyder M, Ridge RD, Copeland J, Stukas AA, Haugen J (1998). Understanding and assessing the motivations of volunteers: a functional approach. J Pers Soc Psychol.

[20] Mousa TY, Freeland-Graves JH (2017). Motivations for volunteers in food rescue nutrition. Public Health.

[21] World Health Organization (2017). Ebola virus disease: fact sheet updated May 2017.

[22] Kpanake L, Tonguino TK, Sorum PC, Mullet E (2018). Duty to provide care to Ebola patients: the perspectives of Guinean lay people and healthcare providers. J Med Ethics.

[23] Topp SM, Price JE, Nanyangwe-Moyo T, Mulenga DM, Dennis ML, Ngunga MM (2015). Motivations for entering and remaining in volunteer service: findings from a mixed-method survey among HIV caregivers in Zambia. Hum Resour Health.

[24] Jigssa HA, Desta BF, Tilahun HA, McCutcheon J, Berman P (2018). Factors contributing to motivation of volunteer community health workers in Ethiopia: the case of four woredas (districts) in Oromia and Tigray regions. Hum Resour Health.

[25] World Health Organization (2017). Guinea: statistics.

[26] Somparé A (2017). La politique et les pratiques de santé en Guinée à l’épreuve de l’épidémie d’Ébola: le cas de la ville de Conakry. Lien social et Politiques.

[27] Rico A, Brody D, Coronado F, Rondy M, Fiebig L, Carcelen A (2016). Epidemiology of epidemic Ebola virus disease in Conakry and surrounding prefectures, Guinea, 2014-2015. Emerg Infect Dis.

[28] Dawes J (2008). Do data characteristics change according to the number of scale points used? An experiment using 5-point, 7-point and 10-point scales. Int J Market Res.

[29] World Health Organization. The Ebola outbreak in Liberia is over. Available: https://afro.who.int/news/ebola-outbreak-liberia-over. Accessed 7 Mar 2019.

[30] La Presse. Ebola: un cordon sanitaire imposé autour de l’épicentre [August 1, 2014]. Available https://www.lapresse.ca/international/afrique/201408/01/01-4788614-ebola-un-cordon-sanitaire-impose-autour-de-lepicentre.php. Accessed 5 Mar 2019.

[31] Fribault M (2015). Ebola en Guinée: violences historiques et régimes de doute. Anthropologie et Santé.

[32] Daily Observer (2014). Fighting Ebola – a test to our patriotism.

[33] Kangethe S (2010). Exploring states of panacea and perfidy of family and community volunteerism in palliative care giving in Kanye CHBC program, Botswana. Indian J Palliat Care.

[34] Gaertner SL, Dovidio JF (2000). Reducing intergroup bias: the common ingroup identity model.

[35] Jack BA, Kirton JA, Birakurataki J, Merriman A (2012). The personal value of being a palliative care community volunteer worker in Uganda: a qualitative study. Palliat Med.

[36] World Health Organization. Engagement communautaire contre la maladie à virus Ebola: des leaders religieux au premier plan pour renforcer les activités de lutte. Available: https://www.afro.who.int/fr/news/engagement-communautaire-contre-la-maladie-virus-ebola-des-leaders-religieux-au-premier-plan. Accessed 7 Mar 2019.

[37] Vareilles G, Marchal B, Kane S, Petrič T, Pictet G (2015). Understanding the motivation and performance of community health volunteers involved in the delivery of health programmes in Kampala, Uganda: a realist evaluation. BMJ Open.

[38] International Labor Organization (2017). Global employment trends for Youth 2017: paths to a better working future.

[39] Claxton-Oldfield S, Claxton-Oldfield J (2007). The impact of volunteering in hospice palliative care. Am J Hosp Palliat Care.

[40] Payne S (2001). The role of volunteers in hospice bereavement support in New Zealand. Palliat Med.

[41] Daoud N, Shtarkshall R, Laufer N, Verbov G, Bar-El H, Abu-Gosh N (2010). What do women gain from volunteering? The experience of lay Arab and Jewish women volunteers in the Women for Women’s Health programme in Israel. Health Soc Care Community.

[42] Muhlenfeld HU (2005). Differences between ‘talking about’ and ‘admitting’ sensitive behaviour in anonymous and non-anonymous web-based interviews. Comput Human Behav.

[43] Lautenschlager GJ, Flaherty FL (1990). Computer administration of questions: more desirable or more social desirability?. J Appl Psychol.

[44] Ormel H, Kok M, Kane S, Ahmed R, Chikaphupha K, Rashid SF (2019). Salaried and voluntary community health workers: exploring how incentives and expectation gaps influence motivation. Hum Resour Health.

